# 

**Published:** 1877-04

**Authors:** 


### Table of Contents.

### ORIGINAL COMMUNICATIONS.

New Intra-Uterine Pessary. *By V. H. Taliaferro, M.D., Atlanta* .. 1

Cerebro-Spinal Fever. *By J. T. Banks, M.D., Griffin, Ga* ......... 4

### REPORTS OF SOCIETIES.

Atlanta Academy of Medicine .................................. 7

### SELECTIONS.

A Ready Test for Arsenical Compounds .......................... 14

Excision of the Rectum for Carcinoma .......................... 17

On the Treatment of Prolapsus Ani in Infants .................. 18

Case Illustrating the Influence of the Imagination upon the Voluntary Muscles ........................ 20

A Case of Bright's Disease of Mechanical Origin; Relief by Operation, 23

The Relation and Hereditary Tendency of Inebriety and Epilepsy.... 25

Bronchitis ................................................. 31

Report of a Case of Idiosyncrasy in Regard to the Effects of Quinine, 36

### EDITORIAL.

Cause of Yellow Fever ....................................... 41

Antiseptic Surgery .......................................... 46

Atlanta Medical College .....................................

Medical Association of Georgia .............................. 47

In Memoriam .................................................

A Case of Paroxysmal Hæmaturia, and Effects of Physostigma in Convulsive Diseases of Children .................................. 48

Osteo-Cartilaginous Deposit in the Knee Joint ................ 49

A Comparison of the Operations for Vesico-Vaginal Fistula ....... 50

American Medical Association .................................

Catalogue of Exchanges ....................................... 51

### EXCERPTA.

Local Uses of Chloral ........................................ 52

Nervousness and Nervines ..................................... 53

The Use of Water to Relieve Pain .............................

On the Immediate Cure of Piles ............................... 54

Choosing a Physician ......................................... 55

A Young Mother ...............................................

Diploma Selling .............................................. 56

A Successful Case of Cæsarian Section .........................

Wood Rendered Incombustible .................................. 58

Lister and the Edinburgh Students .............................

Vaccinnation in Switzerland .................................. 59

Remedies for Chronic Diarrhœa ................................ 60

Kerosene Lamps ............................................... 61

Stimulants Used by the Race ..................................

Chrysophanic Acid ............................................ 63

